# Dynamics of Serum-Neutralizing Antibody Responses in Vaccinees through Multiple Doses of the BNT162b2 Vaccine

**DOI:** 10.3390/vaccines11111720

**Published:** 2023-11-15

**Authors:** Jared Sheehan, Caleb M. Ardizzone, Mayank Khanna, Amber J. Trauth, Michael E. Hagensee, Alistair J. Ramsay

**Affiliations:** 1 Department of Microbiology, Immunology, and Parasitology, Louisiana State University Health Sciences Center, New Orleans, LA 70112, USA; 2 Department of Internal Medicine, Louisiana State University Health Sciences Center, New Orleans, LA 70112, USA

**Keywords:** SARS-CoV-2, spike (S) and receptor-binding domain (RBD), BNT162b2 mRNA vaccine, serum IgG and IgA kinetics, IgG4 subclass response, neutralizing antibodies

## Abstract

SARS-CoV-2 mRNA vaccines are administered as effective prophylactic measures for reducing virus transmission rates and disease severity. To enhance the durability of post-vaccination immunity and combat SARS-CoV-2 variants, boosters have been administered to two-dose vaccinees. However, long-term humoral responses following booster vaccination are not well characterized. A 16-member cohort of healthy SARS-CoV-2 naïve participants were enrolled in this study during a three-dose BNT162b2 vaccine series. Serum samples were collected from vaccinees over 420 days and screened for antigen (Ag)-specific antibody titers, IgG subclass distribution, and neutralizing antibody (nAb) responses. Vaccine boosting restored peak Ag-specific titers with sustained α-RBD IgG and IgA antibody responses when measured at six months post-boost. RBD- and spike-specific IgG4 antibody levels were markedly elevated in three-dose but not two-dose immune sera. Although strong neutralization responses were detected in two- and three-dose vaccine sera, these rapidly decayed to pre-immune levels by four and six months, respectively. While boosters enhanced serum IgG Ab reactivity and nAb responses against variant strains, all variants tested showed resistance to two- and three-dose immune sera. Our data reflect the poor durability of vaccine-induced nAb responses which are a strong predictor of protection from symptomatic SARS-CoV-2 infection. The induction of IgG4-switched humoral responses may permit extended viral persistence via the downregulation of Fc-mediated effector functions.

## 1. Introduction

The rapid development of novel vaccines against severe acute respiratory syndrome coronavirus 2 (SARS-CoV-2) has likely prevented millions of COVID-19-related deaths [1,2]. These include BNT162b2 (Pfizer-BioNTech) and mRNA-1273 (Moderna-NIAID), the first mRNA-based vaccines authorized for human use. The spike (S) glycoprotein became the obvious SARS-CoV-2 vaccine target given its roles in cell attachment and membrane fusion [3]. A primary goal of vaccination is the production of neutralizing antibodies (nAbs), with vaccine-induced nAb responses to the viral receptor-binding domain (RBD) of SARS-CoV-2 expected to inhibit interactions with the human ACE2 receptor. Infection-induced nAb responses in both the blood and respiratory tract are primarily directed against the RBD [4,5,6].

Despite the success of these mRNA vaccines, humoral responses decline by 6–8 months after the two-dose regimen and are less protective against emerging variants [7,8,9,10,11]. Mutations in the RBD, including substitutions at positions K417, E484, and N501, significantly reduced neutralizing activities of immune sera and monoclonal antibody therapeutics [8,12,13]. To enhance the durability of post-vaccination immunity and combat SARS-CoV-2 variants, vaccine boosters (third mRNA dose) were administered to two-dose vaccinees [14]. Initial reports described booster vaccinations as restoring peak antigen (Ag)-specific serum titers, improving nAb responses against emerging variants, preventing new infections, and reducing COVID-19-related mortality [15,16,17,18]. However, long-term humoral responses following boosting are less well characterized. Recent reports describe changes in antigen (Ag)-specific serum IgG subclass profiles [19,20] and an extended half-life for serum nAb responses following mRNA boosting [21,22]. Here, we investigated the dynamics of serum Ag-specific humoral responses through two and three doses of the BNT162b2 mRNA vaccine up to 420 days post-vaccination. 

## 2. Methods

### 2.1. Study Cohort

A 16-member cohort of healthy SARS-CoV-2 naïve participants (aged 25–65, equally distributed by sex) were enrolled during a three-dose monovalent BNT162b2 vaccine series. Serum samples were collected with approval from the Institutional Review Board at Louisiana State University Health Sciences Center, New Orleans, LA (IRB no. 641). All subjects in the cohort stated that they were unaware of previous COVID-19 infection, remained asymptomatic during the course of the study, and were monitored for antibody responses to SARS-CoV-2 nucleocapsid (NC) protein. 

### 2.2. RBD and Spike Ectodomain Protein Expression and Purification

Recombinant Wuhan-Hu-1 RBD and spike (S) ectodomain proteins were expressed from NR-52309 and NR-52421 plasmids, respectively, obtained from BEI Resources (NIAID, Bethesda, MD). For variant RBD constructs, primers were designed to amplify RBD-coding regions from variant full-length spike plasmids (see pseudotyping methods below); PCR products were subcloned into the parent pcDNA3.4 expression vector. RBD- and S-encoding plasmids were transiently transfected into ExpiCHO-S cells (Thermo Fisher, Waltham, MA, USA) following the manufacturer’s protocol. His-tagged RBD and S were purified from clarified culture supernatants via immobilized metal affinity chromatography using HisPur cobalt resin (Thermo Fisher). RBD- and S-loaded resins were washed and eluted using a His-Tag buffer set (GoldBio, St Louis, MO, USA). Elution fractions were concentrated and the buffer exchanged into 1× phosphate-buffered saline (PBS, Crystal City, VA, USA) using Amicon 30 kDa MWCO centrifugal filters (EMD Millipore, Burlington, MA, USA) and stored at −80 °C.

### 2.3. ELISA Detection of Antigen-Specific Antibodies in Serum

Recombinant RBD and S ectodomain proteins were diluted to 2 μg/mL (100 ng/well) in PBS and passively immobilized on 96-well high binding assay plates (Fisher Scientific) overnight at 4 °C. Wells were then washed to remove unbound Ag and blocked with 0.1 mL/well blocking buffer (2% milk/1 × PBS with 0.05% Tween-20). Sera were serially diluted 1:3 (starting at 1:60) in blocking buffer and incubated in Ag-coated plates for one hour at room temperature with shaking at 450 rpm. Assay plates were washed 5× and binding events were detected using HRP-labeled goat α-human IgG(H+L) and goat α-human IgA (1:4000, Thermo Fisher). For IgG subclass-specific assays, binding events were detected using HRP-labeled mouse α-human IgG1 Fc, IgG2 Fc, IgG3 hinge, and IgG4 Fc secondary antibodies (1:2000, Southern Biotech, Birmingham, AL, USA). Plates were developed using KPL SureBlue TMB peroxidase substrate and stop solution (SeraCare, Milford, MA, USA) and the optical density at 450 nm was recorded. Sera were tested for the presence of antibodies against SARS-CoV-2 nucleocapsid (NC) protein as described elsewhere [23]. 

### 2.4. Spike Pseudotyped Virus Production and Neutralization

Lenti-X 293T cells (Takara Bio, Kusatsu, Japan) were cultured in Dulbecco’s modified Eagle’s medium (DMEM) supplemented with 10% fetal bovine serum (FBS), 10 mM HEPES, 1 mM sodium pyruvate, 1× MEM non-essential amino acids, and 1% penicillin/streptomycin in T75 tissue culture flasks (Falcon, Corning, NY, USA) in a humidified 37 °C 5% CO_2_ incubator. At 50–80% confluency, cell monolayers were transfected with 0.53 μg of pcDNA3.4-vectored spike-encoding plasmid, 9.2 μg of pHR’CMV-Luc (VRC5601, NIH Vaccine Research Center), 9.2 μg of pCMV ΔR8.2 (VRC5602), and 0.16 μg of TMPRSS2-encoding plasmid (VRC9260). These four plasmids were delivered using jetPRIME transfection reagent (Polyplus, New York, NY, USA) according to the manufacturer’s protocol. At 96 h post-transfection, culture supernatants were harvested, clarified, and concentrated using Lenti-X Concentrator solution (Takara Bio) according to the manufacturer’s protocol. Concentrated pseudovirus lots were stored at −80 °C.

Plasmids encoding spike from the ancestral Wuhan-Hu-1 strain (Genbank no. NC_045512) and the B.1.351 (GISAID accession number EPI_ISL_1046568), B.1.617.2 (EPI_ISL_3431049), B.1.621 (EPI_ISL_4079202), and B.1.1.529 (EPI_ISL_7801276; GISAID database denoted as BA.1.1.14) SARS-CoV-2 variants were created via gene synthesis (GenScript, Piscataway, NJ, USA) and expressed from the pcDNA3.4 mammalian expression vector. These spike genes were modified in two ways: (1) the native viral leader sequence was replaced with a human CD5 leader sequence and (2) the native cytoplasmic domain was replaced with a truncated HIV-1-related sequence [24]. 

Permissive HEK-293T-ACE2 cells (BEI Resources, NR-52511) were seeded at 3.0 × 10^5^ cells/well in 96-well black wall clear-bottom assay plates (Corning). Sera were serially diluted 1:3 in culture medium (starting at a 1:30 dilution) in sterile 96-well round-bottom tissue culture plates (Corning). SARS-CoV-2 spike pseudovirus was added 1:1 (*v*/*v*) to diluted sera and incubated at 37 °C for one hour. Permissive cell seeding volumes were aspirated from the assay plates and the pseudovirus and serum mixtures were transferred to appropriate assay wells. At 24 h post-transduction, all assay wells were replenished with fresh culture medium (0.2 mL/well). Assay plates were developed at 72 h post-transduction using the ONE-Glo Ex Luciferase Assay Kit (Promega, Madison, WI, USA) according to the manufacturer’s protocol. Luciferase reporter signaling events were recorded using the Cytation1 multi-mode reader (Agilent BioTek, Santa Clara, CA, USA).

### 2.5. Statistics

GraphPad Prism (version 9.5.1) was used for statistical analyses as specified in figure legends. We used two-way ANOVA with Tukey’s multiple comparisons of the mean area under the curve to compare RBD-specific and neutralizing antibody responses and Pearson correlation coefficients for linear correlations. 

## 3. Results

We followed our cohort longitudinally from the baseline through multiple post-vaccination time points and analyzed serum samples for spike RBD-specific antibody responses and nAbs. Demographic information for the cohort is shown in Table 1 along with a summary of ELISA and nAb levels at different time points during the study.

### 3.1. Vaccination Induces Durable RBD-Specific Humoral Responses in Serum

**Figure 1:**
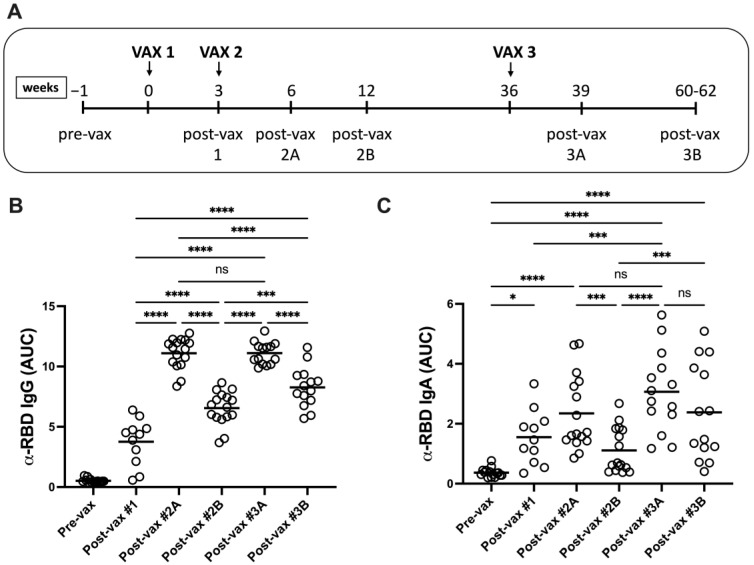
Vaccination induces durable SARS-CoV-2 spike RBD-specific serum IgG and IgA responses. The time course of BNT162b2 vaccine delivery and serum collection is shown (**A**). Receptor-binding domain (RBD)-reactive IgG (**B**) and IgA (**C**) levels (Wuhan-Hu-1) were measured by ELISA as described in Methods. Statistical significance was determined using two-way ANOVA with Tukey’s multiple comparisons of mean area under the curve (AUC) values at each sampling point. * *p* < 0.05, *** *p* < 0.001, **** *p* < 0.0001. ns, not significant.

Vaccinees donated sera prior to vaccination (pre-vax), three weeks following the initial dose (post-vax #1), three weeks and four months after the second dose (post-vax #2A and #2B, respectively), and three weeks and six months following the third dose (post-vax #3A and #3B, respectively) (Figure 1A). 

To measure antigen (Ag)-specific humoral responses, sera were screened against recombinant SARS-CoV-2 spike receptor-binding domain (RBD) protein (Wuhan-Hu-1) by ELISA. Significant increases in α-RBD IgG levels were detected in vaccinee sera after the first and second vaccine doses (Figure 1B). Serum α-RBD IgG titers peaked at three weeks following the second dose with titers declining by four months. Vaccine boosters restored peak serum α-RBD IgG titers and induced sustained RBD-reactive humoral responses at six months after the third dose. Increasing serum α-RBD IgA titers were found through two vaccine doses and declined by four months following the second dose (Figure 1C). Vaccine boosters restored peak α-RBD IgA titers which persisted through six months. Thus, Ag-specific IgG and IgA levels declined but were readily detected in sera for several months after the most recent vaccine dose. 

### 3.2. Booster Vaccination Induces IgG4-Switched Ag-Specific Responses in Serum

**Figure 2:**
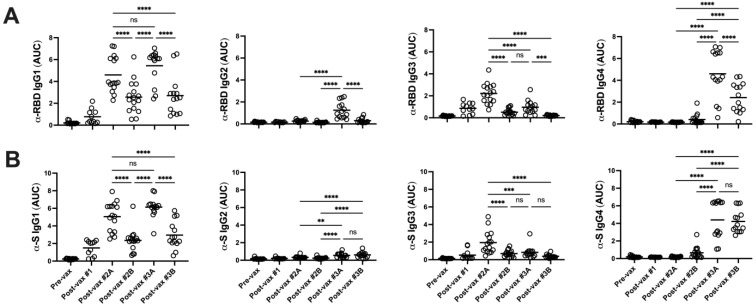
Booster vaccinations induce IgG4-switched Ag-specific responses in serum. RBD-reactive (**A**) and spike-reactive (**B**) IgG subclass responses (Wuhan-Hu-1) were measured by ELISA as described in Methods. Statistical significance was determined as in Figure 1. For clarity, statistical comparisons only for post-vax time points 2A and later are shown. ** *p* < 0.01, *** *p* < 0.001, **** *p* < 0.0001. ns, not significant.

The three-dose vaccine series induced significant changes in RBD-reactive serum IgG subclass distribution. Following the second dose, IgG1 and IgG3 subclasses were predominant while IgG2 and IgG4 levels were negligible (Figure 2A). IgG1 and IgG3 levels declined by four months after the second dose, consistent with Ag-specific IgG titers. Booster vaccination restored RBD-reactive IgG1 levels along with significant increases in both α-RBD IgG2 and IgG4 titers. To confirm these findings, serum IgG subclass titers against the S ectodomain were measured, with similar profiles detected (Figure 2B). While RBD- and S-reactive IgG2 and IgG3 responses were not detected at six months post-boost, significant levels of Ag-specific IgG1 and IgG4 antibodies persisted at this late sampling point. These mRNA vaccine-driven changes in Ag-specific IgG subclass profiles suggest that repeated Ag delivery promoted ongoing class-switch recombination in memory B cells towards distal IgG2- and IgG4-encoding γ heavy chain genes.

### 3.3. Vaccination Elicits Potent but Transient Neutralizing Antibody Responses in Serum

**Figure 3:**
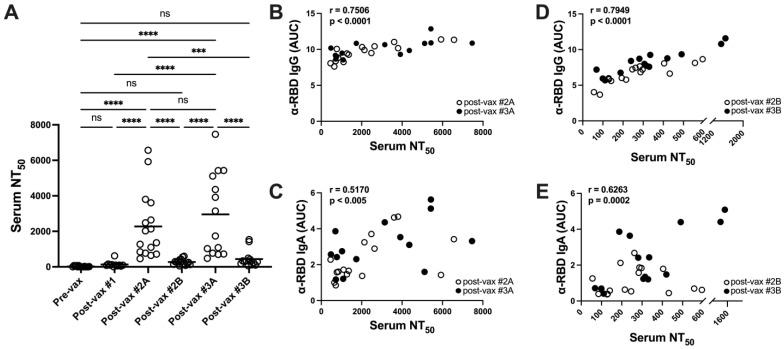
Vaccination elicits potent but transient neutralizing antibody responses in serum. Neutralizing antibody responses were evaluated in SARS-CoV-2 spike pseudotyped virus inhibition assays (**A**) as described in Methods. Statistical significance was determined using two-way ANOVA with Tukey’s multiple comparisons of mean NT_50_ values at each sampling point: *** *p* = 0.003, **** *p* < 0.0001. Correlations between serum NT_50_ values and RBD-reactive IgG or IgA levels were determined at post-vax time points 2A and 2B (**B**,**C**) and post-boosting at time points 3A and 3B (**D**,**E**); Pearson coefficients of correlation (r) are listed on each graph. ns, not significant.

Next, sera were evaluated in SARS-CoV-2 spike (Wuhan-Hu-1) pseudotyped virus inhibition assays to detect nAb responses. Mean serum 50% neutralization titer (NT_50_) values increased significantly after two vaccine doses although with some heterogeneity (1:450 to 1:6500) but had decayed to pre-immune levels at four months (Figure 3A). A single vaccine dose did not establish substantial neutralization activity. Boosters rescued peak NT_50_ values with a similarly wide distribution of neutralization activities but these declined to pre-immune levels by six months following the third vaccine dose. 

Serum NT_50_ values correlated strongly with peak α-RBD IgG titers but only moderately with α-RBD IgA levels (Figure 3B,C). Similarly, the decaying serum NT_50_ values correlated strongly with RBD-reactive IgG and moderately with α-RBD IgA levels at these sampling points (Figure 3D,E). The rapid decay of serum-neutralizing activities suggests a transient character for vaccine-induced circulating nAb responses.

### 3.4. SARS-CoV-2 Variants Are Resistant to Vaccine-Induced Serum α-RBD Reactivity and Neutralizing Antibody Responses

**Figure 4:**
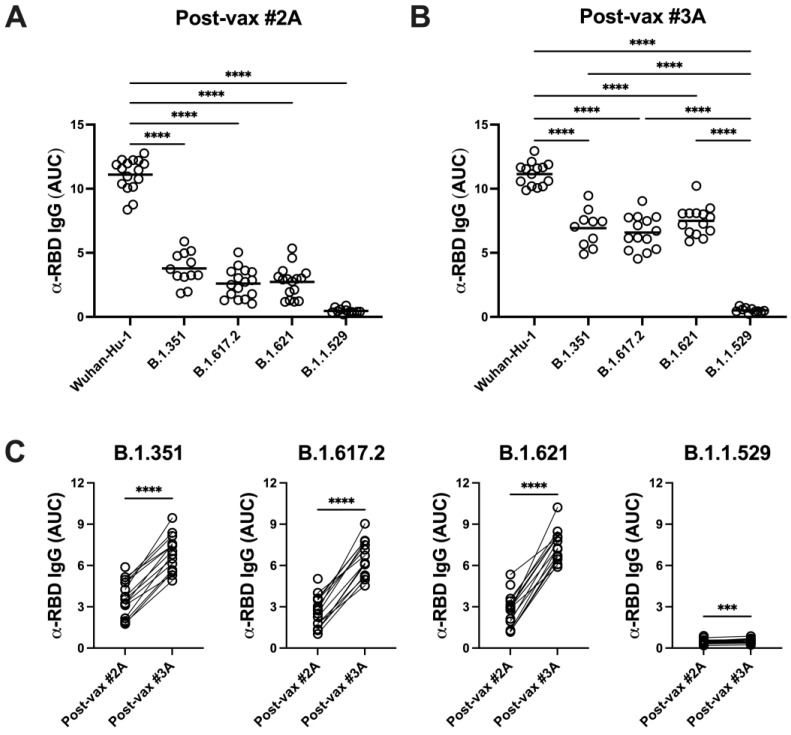
BNT162b2-induced serum IgG responses show impaired reactivity against variant SARS-CoV-2 spike RBD proteins that is improved by vaccine boosting. IgG levels against variant SARS-CoV-2 spike RBD proteins were measured by ELISA at time point 2A (**A**,**C**) and post-boosting at time point 3A (**B**,**C**) as described in Methods. Statistical significance was determined as in Figure 3. **** *p* < 0.0001. Reactivity for each variant at time points 2A and 3A was compared using paired *t*-tests. *** *p* = 0.0003, **** *p* < 0.0001.

To assess the impact of SARS-CoV-2 variants on vaccine-induced humoral immunity, sera collected at time points showing peak RBD-specific IgG titers (post-vax #2A and #3A) were rescreened in binding assays using recombinant RBD proteins from B.1.351 (Beta), B.1.617.2 (Delta), B.1.621 (Mu), or B.1.1.529 (Omicron) variants. Compared with Wuhan-Hu-1 RBD binding, vaccinee serum IgG reactivity declined significantly against each of the variant RBD proteins (Figure 4A,B). Notably, however, booster vaccination significantly improved RBD-reactive IgG titers against all variant RBD proteins (Figure 4C).

**Figure 5:**
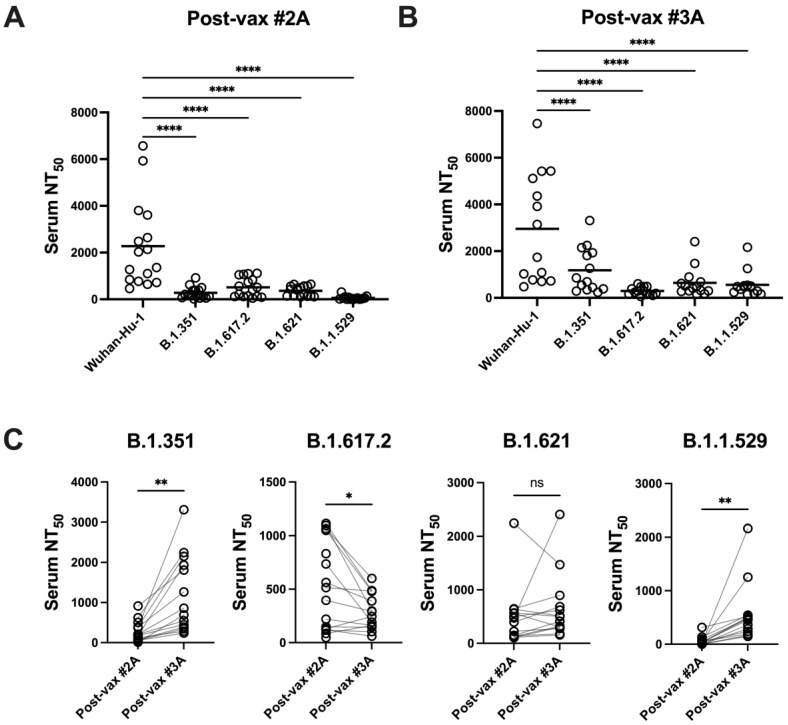
SARS-CoV-2 variants are resistant to BNT162b2-induced neutralizing antibody responses but neutralization activity is improved by vaccine boosting. Neutralizing antibody responses against SARS-CoV-2 variants at time points 2A (**A**,**C**) and post-boosting at time point 3A (**B**,**C**) were evaluated in SARS-CoV-2 spike pseudotyped virus inhibition assays as described in Methods. Statistical significance was determined as in Figure 3. **** *p* < 0.0001. Neutralization activity for each variant at time points 2A and 3A was compared using paired t tests. * *p* < 0.05, ** *p* < 0.01. ns, not significant.

Vaccinee sera were also screened in inhibition assays using variant spike pseudotyped viruses to evaluate the impact of SARS-CoV-2 evolution on BNT162b-induced nAb responses. Sera with peak nAb responses against Wuhan-Hu-1 (post-vax #2A and #3A) showed significantly reduced neutralization activity against all variant pseudoviruses (Figure 5A,B). Vaccine boosters enhanced serum neutralization activity against B.1.351 and B.1.1.529 spike pseudoviruses but activity against B.1.617.2 variant spike pseudovirus declined significantly following the third vaccine dose. Decreases observed in both serum IgG responses and neutralization activity against this panel of variants indicate that vaccine-elicited humoral responses were sensitive to ongoing SARS-CoV-2 evolution.

## 4. Discussion

This report describes longitudinal analyses of serum antibody responses in a three-dose BNT162b2 mRNA vaccinee cohort. High and sustained levels of serum RBD-specific IgG and IgA were detected following two and three vaccine doses. While Ag-specific antibody levels peaked at three weeks following the second dose, α-RBD IgG and IgA titers declined by four months. The detection of vaccine-induced Ag-specific IgA is noteworthy as this isotype has been reported to dominate the early serum antibody response to SARS-CoV-2 infection [5]. Vaccine boosters restored peak serum RBD-reactive IgG and IgA titers which declined again by six months after this third dose. Despite these declines, α-RBD IgG and IgA remained readily detectable in serum at six months after the third dose. 

In addition to establishing persistent α-RBD IgG titers, serum IgG subclass profiles evolved through the three-dose regimen. The detection of inflammatory IgG1 and IgG3 responses in two-dose sera was expected given previous reports of elevated Ag-specific IgG1 and IgG3 titers during early humoral responses to SARS-CoV-2 infection and vaccination [25,26,27]. The onset of Ag-specific IgG4 antibodies has been documented in cases of repeated Ag exposure in non-infectious settings, including immunotherapy for allergy [28,29]. Coupled with the weakly boosted α-RBD IgG3 titers observed in this cohort, the unexpectedly robust and sustained induction of RBD- and S-reactive serum IgG4 following multiple vaccine doses in the present study is likely a consequence of continuous class-switch recombination (CSR) events in Ag-experienced B cells within germinal centers. The order of the γ heavy chain genes (5′-*Cγ*3-*Cγ*1-*Cγ*2-*Cγ4*-3′) on human chromosome 14 supports a hypothesis of progressive CSR-mediated IgG4 expression in response to a third vaccine dose [29,30,31]. During the course of this study, reports emerged describing similar mRNA vaccine-driven changes in serum Ag-specific IgG subclass profiles [19,20]. Unexpectedly high Ag-specific IgG4 titers in post-boost vaccinees are likely due to a slowly-developing population of IgG4-switched antibody-secreting cells derived from reactivated memory B cells established through the previous two-dose mRNA regimen.

Serum nAb responses, while heterogeneous, peaked at three weeks after the second and third vaccine doses and correlated strongly with RBD-specific IgG titers. Recent serum antibody mapping analyses involving members of this cohort revealed donor-to-donor variability in the breadth of epitope targeting through two and three vaccine doses [23]. These variations in magnitude and breadth of reactivity likely contribute to the diversity of serum neutralization activities against vaccine strain-matched Wuhan-Hu-1 S pseudovirus. Uniform decay of neutralization activity was also observed. Since RBD-specific IgG and IgA titers were seen for several months post-vaccination, the rapid decay of nAb responses suggests that neutralizing classes of serum antibodies represent a minority population of BNT162b2 mRNA vaccine-elicited humoral immunity. Overall, our longitudinal analyses characterize poor durability of BNT162b2-induced serum nAb responses.

The dynamics of immune escape by RNA viruses limits the efficacy of vaccine-induced protective immune responses [32,33]. In particular, mutations within the spike RBD afford viral resistance against neutralizing activities of both therapeutic antibodies and immune sera [34,35]. In the present study, BNT162b2-induced serum IgG antibodies demonstrated significantly reduced reactivity against RBD proteins derived from the B.1.351, B.1.617.2, B.1.621, and B.1.1.529 variants through three vaccine doses. Poor serum IgG reactivity against B.1.1.529 RBD was expected given 16 characterized mutations within this variant domain. While vaccine boosters improved RBD-reactive IgG antibody titers against B.1.351, B.1.617.2, and B.1.621 variant RBD proteins, reactivity against B.1.1.529 RBD remained low, with these titers aligning with the limited neutralization activities found against the same panel of variant S pseudoviruses. Post-vaccination NT_50_ values for variant S pseudoviruses were significantly lower than against vaccine strain-matched Wuhan-Hu-1 S pseudovirus, with the observed decline in serum NT_50_ values for B.1.617.2 S pseudovirus likely due to post-boost narrowing of nAb responses towards variant-specific mutations in the spike glycoprotein [23]. 

While testing for mucosal humoral immunity or T cell responses to BNT162b2 vaccination was beyond the scope of the present study, it is important to note that both may play an important role in host defense against respiratory pathogens. Secretory IgA may prevent SARS-CoV-2 adhesion to target epithelial cells via neutralization of the coronavirus spike glycoprotein [5] although such responses were poorly activated following intramuscular (IM) administration of BNT162b2 vaccine, at least in saliva [36]. Notably, however, intranasal protein boosting following IM delivery of mRNA vaccine-induced respiratory IgA along with local B and T cell activation correlated with protection against SARS-CoV-2 challenge in a murine model [37]. SARS-CoV-2 infection induces vigorous CD4+ and CD8+ T cell responses against different viral antigens while CD4+ T cells are clearly important for the success of COVID-19 vaccines [38]. The preservation of T cell reactivity against ancestral SARS-CoV-2 across variant sequences [39] contrasts with the apparent loss of nAb activity against SARS-CoV-2 variants. 

In summary, longitudinal analysis of the dynamics of BNT162b2-induced humoral immunity through a three-dose mRNA vaccine series over a period of 420 days suggests poor durability of BNT162b2-elicited nAb responses. A potential limitation of our study is the possibility of asymptomatic infection with SARS-CoV-2 that could impact the antibody responses that were detected in our cohort. We did, however, monitor antibody responses against the SARS-CoV-2 viral nucleocapsid protein in the cohort and saw no clear spikes in reactivity during the course of the study. It should also be noted that low-level responses against the nucleocapsid may be a result of SARS-CoV-2 infection but also, potentially, of previous coronavirus infections due to the cross-reactive nature of this viral protein [40]. Our study, albeit in a cohort of limited size, confirms recent reports of the limited durability of specific IgG and IgA responses by ELISA [41] and of nAb responses against variants [21] following mRNA vaccination against SARS-CoV-2. nAb titers are strongly predictive of protection from symptomatic SARS-CoV-2 infection; the rapid decay of serum neutralizing activities indicates that this vaccination approach may not be optimal for long-term sterilizing immunity [42]. Vaccine efficacy is further limited through the evolution of diverse Omicron-related subvariants which demonstrate increased transmission rates and potential for immune escape [43,44]. In addition to transient nAb responses and ongoing viral evolution, the efficacy of mRNA vaccine-induced humoral immunity may be restricted through elevated levels of Ag-specific IgG4 following vaccine boosting. Since current and future variants are expected to show resistance to vaccine-elicited nAb activities, high IgG4 antibody titers may facilitate extended viral persistence during future SARS-CoV-2 infections as Fc-mediated effector functions are downregulated during IgG4-switched responses [29]. In contrast, a cohort receiving the adenovirus vectored AZD1222 vaccine did not develop long-term IgG4 responses [20]. We conclude that multivalent formulations covering updated variant strains and heterologous vaccine combinations [45,46] should be considered for induction of polyfunctional and protective immune responses in future vaccination campaigns against SARS-CoV-2 and other viral pathogens.

## 5. Conclusions

In a longitudinal study of the dynamics of BNT162b2-induced humoral immunity through a three-dose mRNA vaccine regimen, we have demonstrated that while vaccine boosting restored peak α-RBD IgG and IgA antibody titers and generated strong antiviral neutralization responses, these nevertheless decayed to pre-immune levels within six months. Boosters enhanced serum reactivity and nAb responses against Beta, Delta, Mu, and Omicron SARS-CoV-2 strains; however, all variants that were tested showed resistance to two and three vaccine-dose immune sera. Our data indicate poor durability of vaccine-induced nAb responses while the induction of markedly elevated levels of antibodies of the IgG4 subclass could potentially facilitate extended viral persistence via downregulation of Fc-mediated effector functions. 

## Figures and Tables

**Table 1:** Cohort demographics and summary of antibody levels. Demographics are shown and serum IgG and neutralization titers are summarized across the collection period. Antigen-reactive antibody titers were categorized as below the limit of detection (<1:40), low (>1:40 and <1:160), medium (>1:160 and <1:1280), and high (>1:1280). Significant pairwise comparisons across vaccination points are denoted ^A^ 1st vax vs. 2nd vax, ^B^ 1st vax vs. 3rd vax (3A) and ^D^ 3rd vax (3A) vs. 3rd vax (3B). Significance values are denoted ** *p* < 0.01, *** *p* < 0.001 and **** *p* < 0.0001. 2A, 3A, and 3B correspond to sample collection time points denoted in Figure 1A.

Demographics (*n* = 16)		Baseline	1st Vax	2nd Vax (2A)	3rd Vax (3A)	3rd Vax (3B)
**Age|Mean (SD)**		45 (13.6)	-	-	-	-
Sex|Male (Female)		9 (7)	-	-	-	-
Race|*n*		W (15), H (1)	-	-	-	-
Vaccination		-	February 2021	February 2021	October–December 2021	-
Sample Collection		May2020–January 2021	March–April 2021	March–April 2021	November 2021	April 2022
**Antibody**						
Spike RBD IgG Titer A **, B ****, C ****, D ***		*n* (%)				
	Below LOD	13 (81.25%)	1 (6.25%)	0	0	0
	Low	1 (6.25%)	2 (12.5%)	0	0	0
	Medium	2 (12.5%)	13 (81.25%)	7 (43.75%)	0	0
	High	0	0	9 (56.25%)	16 (100%)	16 (100%)
NC IgG Titer		*n (%)*				
	Below LOD	12 (75%)	13 (81.25%)	12 (75%)	12 (75%)	12 (75%)
	Low	4 (25%)	3 (18.75%)	3 (18.75%)	3 (18.75%)	3 (18.75%)
	Medium	0	0	1 (6.25%)	1 (6.25%)	1 (6.25%)
	High	0	0	0	0	0
Neutralization ^A****, B****, D****^						
	Serum NT_50_|mean (SD)	20.31 (29.14)	133.33 (166.49)	2272.11 (1862.06)	2955.08 (2316.72)	438.51 (456.66)

Abbreviations: Vax, vaccination; SD, standard deviation; LOD, limit of detection; W, White; H, Hispanic.

## Data Availability

Raw data will be available upon suitable request to the corresponding author.
